# Classification of plug seedling quality by improved convolutional neural network with an attention mechanism

**DOI:** 10.3389/fpls.2022.967706

**Published:** 2022-08-04

**Authors:** Xinwu Du, Laiqiang Si, Xin Jin, Pengfei Li, Zhihao Yun, Kaihang Gao

**Affiliations:** ^1^College of Agricultural Equipment Engineering, Henan University of Science and Technology, Luoyang, China; ^2^Science & Technology Innovation Center for Completed Set Equipment, Longmen Laboratory, Luoyang, China; ^3^Collaborative Innovation Center of Machinery Equipment Advanced Manufacturing of Henan Province, Luoyang, China

**Keywords:** plug seedlings, convolutional neural network, EfficientNet-B7-CBAM model, transfer learning, quality classification

## Abstract

The classification of plug seedling quality plays an active role in enhancing the quality of seedlings. The EfficientNet-B7-CBAM model, an improved convolutional neural network (CNN) model, was proposed to improve classification efficiency and reduce high cost. To ensure that the EfficientNet-B7 model simultaneously learns crucial channel and spatial location information, the convolutional block attention module (CBAM) has been incorporated. To improve the model’s ability to generalize, a transfer learning strategy and Adam optimization algorithm were introduced. A system for image acquisition collected 8,109 images of pepper plug seedlings, and data augmentation techniques improved the resulting data set. The proposed EfficientNet-B7-CBAM model achieved an average accuracy of 97.99% on the test set, 7.32% higher than before the improvement. Under the same experimental conditions, the classification accuracy increased by 8.88–20.05% to classical network models such as AlexNet, VGG16, InceptionV3, ResNet50, and DenseNet121. The proposed method had high accuracy in the plug seedling quality classification task. It was well-adapted to numerous types of plug seedlings, providing a reference for developing a fast and accurate algorithm for plug seedling quality classification.

## Introduction

China is the world’s leading producer of vegetables. China’s vegetable cultivated area sowing area in 2020 was 2148.54 × 10^4^ hm^2^, and its production was 74912.90 × 10^4^ t (Meng et al., 2021; Zhang et al., 2022). In order to meet the increasing demand for vegetable planting and ensure a safe and efficient supply of seedlings, vegetable seedling production has adopted an intensive plug seedling cultivation method which is characterized by a high survival rate, low labor costs, and convenient transportation. Approximately 60% of the world’s vegetable varieties currently use plug seedling technology (Li et al., 2021; Shao et al., 2021; Han et al., 2022). The plug seedlings enhanced the quality of vegetable seedlings as a whole. However, due to the sowing accuracy, seed quality, and seedling environment, the nursery tray contained empty plug cells, seedlings with poor growing conditions, and dead seedlings. About 5–10% of the total number of seedlings were comprised of these empty plug cells and weak seedlings. If they are not eradicated, they will not only cause economic losses but also hinder future machine transplantation operations. For the quality of plug seedlings, it is necessary to remove empty plug cells and weak seedlings from the tray cells and replant them with strong seedlings (Jin et al., 2020; Wen et al., 2021; Yang et al., 2021).

In intensive plug seedlings, classification of seedling quality is necessary to ensure overall seedling quality. Currently, this process relies heavily on manual labor. Manual classification is time-consuming, laborious, inefficient, and prone to error, making it challenging to meet the demands of large-scale seedling production. Consequently, it is essential to investigate the automated plug seedling quality classification technology, and machine vision is a crucial component of this technology (He et al., 2019; Yang et al., 2020; Tong et al., 2021). Early identification of plug seedlings using machine vision and conventional image processing techniques. Tong et al. (2018) presented a skewness correction algorithm for images of plug seedlings based on the canny operator and hough transform. The method is based on the watershed algorithm and the center of gravity method to extract leaf area and seedling leaf number from images of plug seedlings for quality evaluation; the results showed that the average accuracy of empty plug cells and weak seedlings reached 98%. Wang et al. (2018) developed a device for automatically supplementing vegetable plug seedlings to obtain accurate information about plug seedlings. By obtaining information on the vegetation statistics values of each cell, the method achieved a 100% accurate classification of plug cells and seedling cells. Jin et al. (2021) proposed a computer vision-based architecture to identify seedlings accurately. The method extracts leaf area information from plug seedlings using a genetic algorithm and a three-dimensional block matching algorithm with optimal threshold segmentation. Based on the results, the detection accuracy for healthy seedlings reached 94.33%. Wang et al. (2021) proposed a non-destructive monitoring method for the growth process of plug seedlings based on a Kinect camera, which determines the germination rate in trays by reconstructing leaf area analysis with an error of less than 1.56%. To determine the growth status of plug seedlings, the primary research used the threshold pre-processing method for threshold segmentation and statistical pixel value information. The technology is relatively mature. Nonetheless, the following problems remain. (1) Following segmentation, seedling growth data is lost. (2) To obtain the proper segmentation threshold, a large number of human tuning parameters are required. (3) More complex algorithms must be developed to increase the precision of leaf area segmentation.

The application of deep CNN models in agriculture has achieved significant results in recent years, including disease detection (Sharma et al., 2022), weed identification (Wang et al., 2022), and crop condition monitoring (Zhao L. et al., 2021; Tan et al., 2022). Using deep learning techniques to classify the quality of plug seedlings can better meet the development requirements of seedling production. Namin et al. (2018) proposed a robust AlexNet-CNN-LSTM architecture for classifying the various growth states of plants. This method improved model performance by embedding long short-term memory network (LSTM) units and achieved 93% recognition accuracy by reducing model parameters. Xiao et al. (2019) developed a transfer learning CNN for the plug seedling classification model. Based on a limited sample of empty plug cells, weak seedlings, and strong seedlings, the final classification accuracy was 95.50%. Perugachi-Diaz et al. (2021) used an AlexNet network to predict the growth of cabbage seedlings. According to the results, the method provided a reliable and effective classification of cabbage seedlings with an optimal recognition accuracy of 94%. Garbouge et al. (2021) proposed a method for tracking the growth of seedlings that combines RGB with deep learning. As a result of the method, seedlings grown in plug cells, seedlings at the cotyledon stage, and seedlings at the true leaf stage performed with an average classification accuracy of 94%. Compared to other models discussed in the paper, Kolhar and Jagtap (2021) proposed a CNN-ConvLSTM-based model for seedling quality classification of Arabidopsis thaliana that achieved 97.97% classification accuracy with very few trainable parameters. According to the appeal study, CNNs had a higher accuracy rate and more excellent stability than conventional image processing methods without requiring threshold segmentation. However, the following issues persist: (1) The majority of current CNN have high computational complexity and a large number of parameters, making it difficult to directly deploy and apply them in this paper’s quality classification of plug seedlings. (2) Due to the variability between different task goals, CNN models required a certain amount of target data for adaptive learning. Constructing the desired data set required much human time and effort.

Using pepper plug seedlings as the research object, a new and more effective CNN model, EfficientNet-B7-CBAM, is presented for seedling quality classification. Following is a summary of the main contributions and innovations.

1. A classification standard for various qualities of plug seedlings is developed. On the basis of this standard, an 8109-image dataset of plug seedlings is compiled to aid in developing a plug seedling quality classification model.

2. A novel attention-based recognition model for plug seedling quality classification, the EfficientNet-B7-CBAM model, is proposed. By deeply integrating the CBAM module and the EfficientNet-B7 model, the model can simultaneously acquire feature channel information and spatial information attention and enhance the model’s ability to learn important information about the plug seedling region.
3. The lightweight and high-performance EfficientNet-B7-CBAM model can provide technical support for developing the automated classification of plug seedling quality equipment.

## Experimental data

### Data acquisition

**FIGURE 1:**
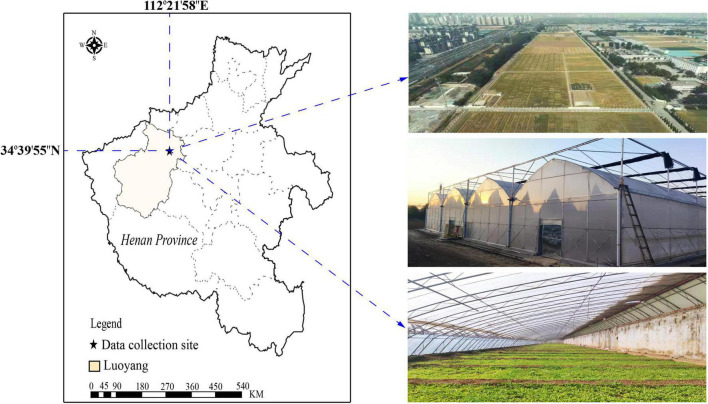
Location of data collecting and multi-span seedling greenhouses.

Pepper plug seedlings were grown from Oct to Nov 2021 in a multi-span seedling nursery at the Academy of Agricultural Sciences, Luoyang City, Henan Province, China (34°39′55″N, 112°21′58″E), as shown in Figure 1. The temperature in the greenhouse was kept between 20–25°C during the day and 10–15°C at night. The chosen pepper variety was the stress-resistant Luo Jiao 308 variety. The seeds were sterilized before being sown with a single hole and seed. Approximately 9,000 pepper seeds were planted in 540 mm × 280 mm, 32-cell nursery trays that contained a mixture of peat, vermiculite, and perlite (at a 3:1:1 ratio).

**FIGURE 2:**
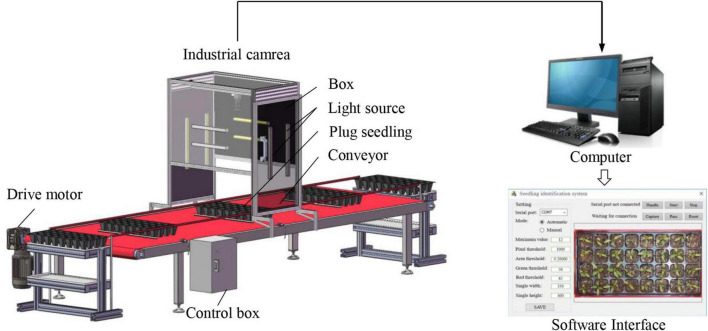
Picture capture system.

Image the tops of pepper plug seedlings using the selected data acquisition equipment Hikvision MV-CE200-10UC color sensor camera with a frame rate of 14 fps and a resolution of 5472 × 3648 pixels. The USB3.0 port connects the camera to the computer. The lens was the MVL-HF1224M-10MP model with a focal length of 12 mm. When shooting, the camera was mounted vertically above the nursery trays at the height of H = 545.4 mm, effectively encompassing the standard nursery trays area. Three light-emitting diodes (LED) with a power of 5.76 W/m were installed on the inner wall of the lightbox to supplement the light during image capture, thereby enabling the camera to capture the fine details of the seedlings in the nursery trays. The image capture system is shown in Figure 2.

### Data preprocessing

**FIGURE 3:**
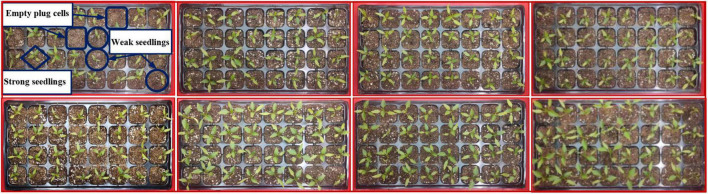
Sample of pepper seedlings growing for 21 days.

Pepper seedlings at approximately 21 days after emergence are shown in Figure 3. Within the same batch of pepper plug seedlings, there are empty plug cells caused by non-germinating seeds, weak seedlings with slow growth, and strong seedlings for transplantation due to biological differences between individual seedlings. Leaf area characteristics were obtained to classify three distinct types of plug seedlings with varying qualities to construct image data sets of empty plug cells, weak seedlings, and strong seedlings.

**FIGURE 4:**
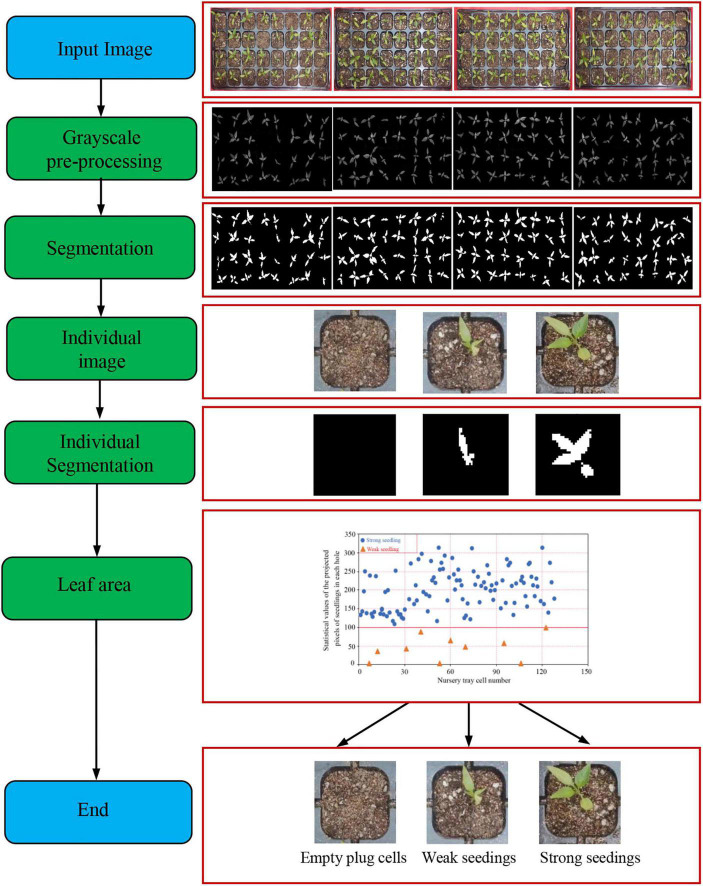
The image processing flow of the leaf area of pepper seedlings.

Leaf area is a popular gauge employed in agricultural cultivation and breeding techniques, and it is one of the most important indicators for determining crop yield and quality. For the purpose of categorizing the quality of pepper seedlings, leaf area parameters were extracted from pepper seedlings. The leaf area extraction procedure for pepper seedlings is shown in Figure 4.

**FIGURE 5:**
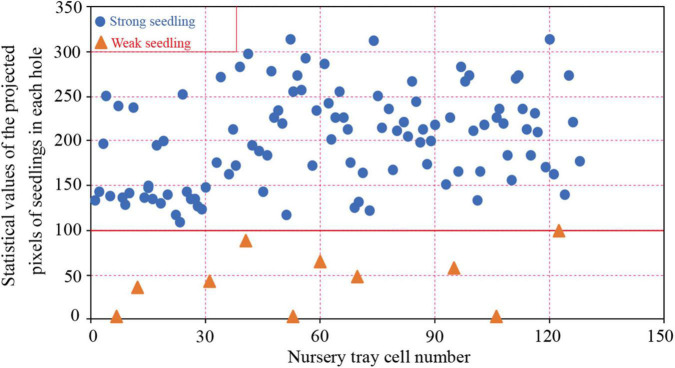
Pixel statistical scatter plot of seedling leaf area.

The distribution of pixel values for the leaf area of 21-day-old pepper plug seedlings is shown in Figure 5. Leaf areas were 0 in empty plug cells, less than 100 in weak seedlings, and at least 100 in strong seedlings.

**FIGURE 6:**
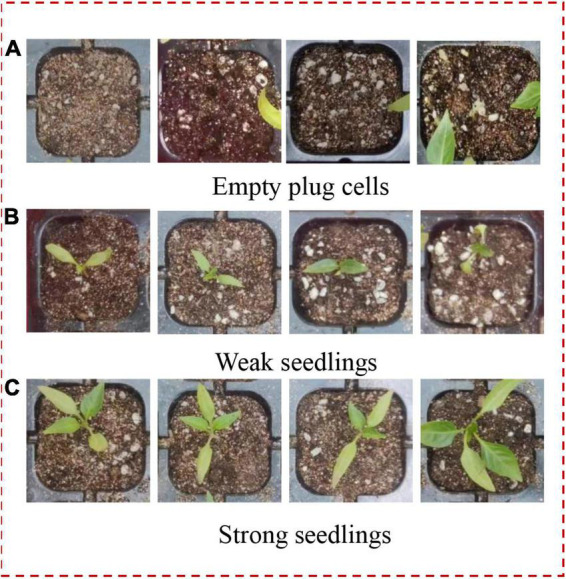
Plug seedlings of different qualities. **(A)** Empty plug cells. **(B)** Weak seedlings. **(C)** Strong seedlings.

In order to construct training data set, empty plug cells, weak seedlings, and strong seedlings were extracted from the original RGB image based on their pixel value distributions. Pepper plug seedlings of differing qualities are shown in Figure 6. After the reduction, 2,210 empty plug cells, 3,381 weak seedlings, and 2,518 strong seedlings were obtained. Using the Albumentations library to expand data, the original data for pepper plug seedlings were enhanced to include additional image data. The data were clipped, rotated, and inverted to generate a data set containing 19,603 images, as shown in Table 1.

**TABLE 1:** The sample size of the training set and validation set.

Class	Training dataset	Validation dataset	Total
Strong seedlings	5,042	843	5,885
Weak seedlings	6,230	1,037	7,267
Empty plug cells	5,480	971	6,451

## Methodology

This study chose the lightweight, high-precision, and simple-to-deploy EfficientNet-B7 model as the benchmark network for the application of intelligent recognition algorithms to images of plug seedlings in agriculture. To increase the network model’s recognition accuracy, the CBAM module was introduced to optimize and enhance the EfficientNet-B7 model, which was then renamed EfficientNet-B7-CBAM.

### Efficientnet-B7 network structure

**FIGURE 7:**
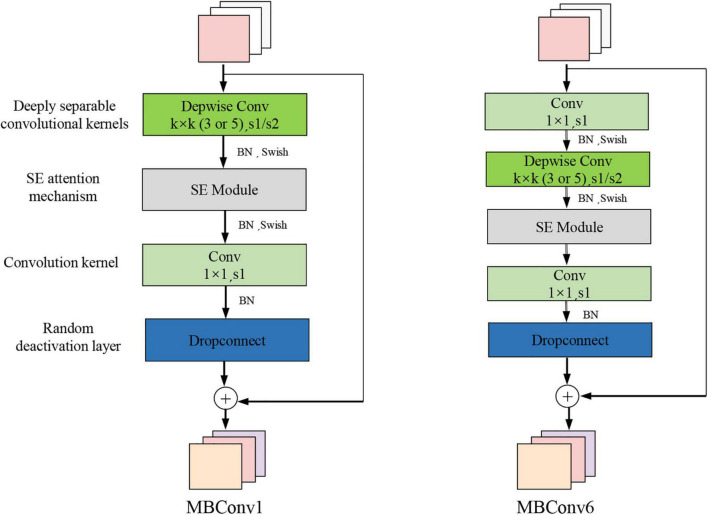
Mobile inverted bottleneck convolution.

**FIGURE 8:**
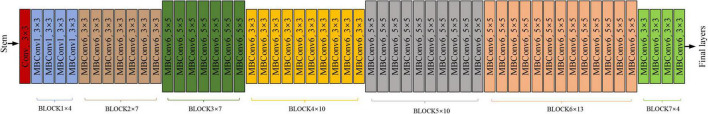
The network structure of EfficientNet-B7.

To improve the performance of the CNN model, we increased the input image’s resolution as well as the network’s depth and width. However, the concurrent use of the three methods may result in severe issues, such as the loss of model gradients and the degradation of models. The emergence of EfficientNet is characterized by a balance between depth, width, and resolution. There were B0-B7 EfficientNet versions. Mobile Inverted Bottleneck Convolution (MBConv) was the core structure of the network (Zhang et al., 2020; Liu et al., 2021; Bhupendra et al., 2022). This module introduces the Squeeze-and-Excitation Network (SE)’s core concept to optimize the Network’s structure, as shown in Figure 7. The MBConv module first uses 1 × 1 convolutions to up-dimension the feature map, followed by *k* × *k* depthwise convolutions. After that, SE modules adjust the feature map matrix, and eventually, 1 × 1 convolutions to down-dimension the feature map. When the input and output feature maps have the same shape, the MBConv module is also capable of performing short-cut concatenation. This structure reduces model training time. A typical Efficientnet-B7 model consists of 55 layers of MBConvs modules, 2 layers of Convs modules, 1 layer of global average pool, and 1 layer of FC classification. The network architecture of EfficientNet-B7 is shown in Figure 8.

### Convolutional block attention module model

**FIGURE 9:**
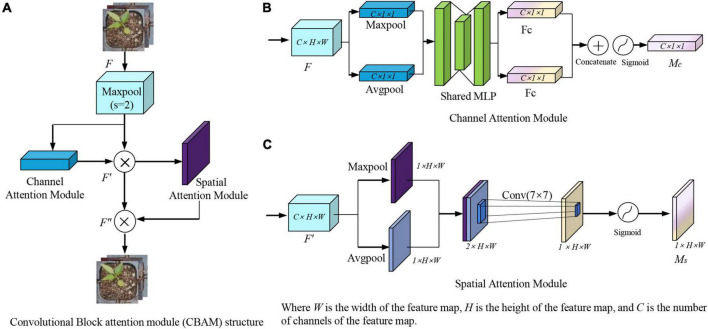
CBAM attention module. **(A)** Convolutional block attention module (CBAM) structure. **(B)** Channel attention module. **(C)** Spatial attention module. Where *W* is the width of the feature map, *H* is the height of the feature map, and *C* is the number of channels of the feature map.

This section will provide an overview of the CBAM attention mechanism. Woo proposed CBAM, which would be comprised of two modules: the channel attention module and the space attention module (Bao et al., 2021; Gao et al., 2021; Zhao Y. et al., 2021). The CBAM module is shown in Figure 9. The CBAM module first generates the feature map *F′ via* the channel attention module, then the feature map *F″ via* the spatial attention module, given a middle layer feature map *F* as input, as shown in Figure 9A. The process of calculation can be expressed as Equation 1.


(1) $$\left\{ \begin{array}{c} F^{\prime }=M_{c}\,\left(F\right)⊗F\\ F^{\prime \prime }=M_{s}\,\left(F^{\prime }\right)⊗F^{\prime } \end{array}\right.$$

where ⊗ represents the multiplication operation between the corresponding elements. *F* (∈ *R*^*C*×*H*×*W*^) represents the input feature map. *M*_*c*_ (∈ *R*^*C*×1×1^) represents the output weight of *F*′ through the channel attention. *M*_*s*_ (∈ *R*^1×*H*×*W*^) represents the output weight of *F″* through the spatial attention.

The module of the channel attention mechanism is shown in Figure 9B. In the first step of the channel attention mechanism, the average pooling and maximum pooling operations are performed based on width and height to generate two layers of *C* × 1 × 1 feature maps. Then, they are fed to the shared MLP layer for summation and activated by the sigmoid to produce the final channel attention feature weights *M*_*c*_. The channel attention calculation procedure can be expressed as Equation 2.


(2) $$M_{c}\left(F\right)=\sigma [MLP\left(AvgPool\left(F\right)\right]+MLP\left[MaxPool\left(F\right)\right]$$

where σ represents a sigmoid function; MLP represents a multilayer perceptron.

The module of the spatial attention mechanism is shown in Figure 9C. As input to the spatial attention mechanism is the feature map *F*′. First, the average pooling and maximum pooling operations are performed on the channel to generate a two-layer 1 × *H* × *W* feature map, which is then subject to the Concatenate operation. The dimension of the feature map is then reduced using a 7 × 7 convolution kernel, and the Sigmoid function is used to generate the spatial attention weights *M*_*s*_. The spatial attention calculation procedure can be expressed as Equation 3.


(3) $$M_{s}\,\left(F\right)=\sigma \,\left\{f^{7\times 7}\,\left[A\,v\,g\,P\,o\,o\,l\,\left(M\,c\right);M\,a\,x\,P\,o\,o\,l\,\left(M\,a\,c\right)\right]\right\}$$

Where *f*^7×7^ is the convolution operation with a convolution kernel size of 7 × 7, which is used to extract the spatial features of the target.

### EfficientNet-B7-CBAM model

**FIGURE 10:**
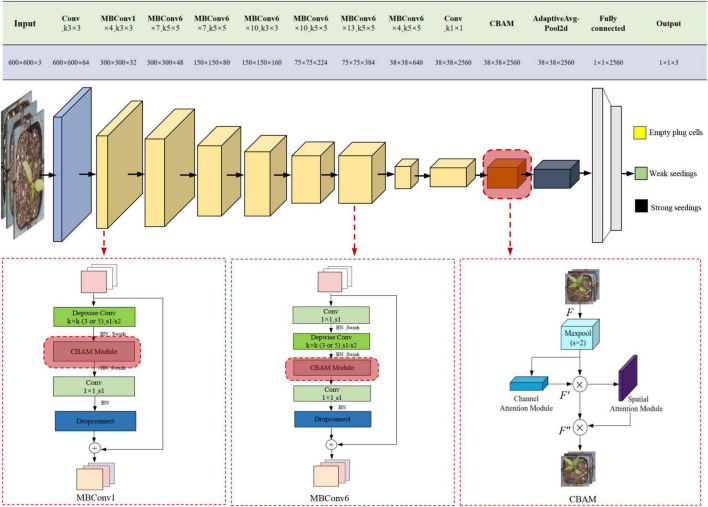
EfficientNet-B7-CBAM model.

EfficientNet-B7 is composed of the MBConv stack, with each MBConv module containing a SE module. The SE module controls the focus or gating of channel dimensions. The model can emphasize the channel characteristics that contain the most information while ignoring the channel characteristics that are unimportant. However, this operation only considered the information of the channels and lost the spatial information, which played a crucial role in the visual recognition task of seedlings, which negatively impacted the classification performance of seedlings. CBAM was added to Efficientnet-B7 in this study to improve the model’s ability to extract features. The improved EfficientNet-B7-CBAM network structure is shown in Figure 10. The following enhancements have been made relative to the original Efficientnet-B7 network model:

(1) The SE module within each MBConv module of the original EfficientNet-B7 model was replaced with a CBAM module. This allowed the network to acquire channel information without losing crucial spatial information regarding the pepper plug seedlings.
(2) The CBAM module was embedded in the EfficientNet-B7-CBAM model after the second convolutional layer. It improved the model’s ability to classify different quality plug seedlings by refining the extracted feature information and enhancing the model’s classification capability.

### Adam optimization algorithm

A classical optimization algorithm is used to optimize the EfficientNet-B7 model: Stochastic Gradient Descent (SGD). Due to the same learning rate for each parameter, it was difficult to obtain a suitable learning rate for the SGD algorithm. In addition, the SGD optimization algorithm converges rapidly to a local optimum when training the model, which causes the model to be unable to obtain an optimal training model when performing different quality pepper plug seedling classification tasks. In order to solve the above problem, this paper employed the Adam optimization algorithm. Each parameter of the Adam algorithm maintained a learning rate and was adjusted individually as a result of training. Additionally, each learning rate adjustment was bias-corrected in order to reduce the fluctuations in parameter updates and enhance the smoothness of the model convergence. In the Adam optimization algorithm, momentum updates are combined with learning rate adjustments, and the learning rates of each parameter are dynamically adjusted by the first and second moments of the gradient (Yu and Liu, 2019; Ilboudo et al., 2020; Cheng et al., 2021). The calculation process can be expressed as Equation 4.


(4) $$\left\{ \begin{array}{c} \theta_{t}=\theta_{t-1}-\alpha \cdot \frac{{\hat{m}}_{t}}{\sqrt{{\hat{v}}_{t}}+\varepsilon }\\ {\hat{m}}_{t}=\frac{m_{t}}{1-\beta_{1}^{t}}\\ {\hat{v}}_{t}=\frac{v_{t}}{1-\beta_{2}^{t}}\\ m_{t}=\beta_{1}\cdot m_{t-1}+\left(1-\beta_{1}\right)\cdot g_{t}\\ v_{t}=\beta_{2}\cdot v_{t-1}+\left(1-\beta_{2}\right)\cdot g_{t}^{2}\\ g_{t}=\nabla_{\theta }f_{t}\,\left(\theta_{t-1}\right) \end{array}\right.$$

where θ_*t*_ and θ_*t*−1_ represents the parameter values of the *t*th and *t*-1th updates. *m*_*t*_ represents the exponentially shifted mean of the gradient. *v*_*t*_ represents the squared gradient. ${\hat{m}}_{t}$ represents the updated value of *m*_*t*_. ${\hat{v}}_{t}$ represents the updated value of *v*_*t*_. β_1_ and β_2_ represent the constants used to control the exponential decay. *g*_*t*_ represents th first-order derivative. The default values for each of the parameters are: *α* = 0.001, *β*_1_ = 0.9, *β*_2_ = 0.999, and *ε* = 10^–8^.

### Transfer learning

Given that images from different domains contain common underlying features among them, transfer learning makes the training more stable by transferring knowledge of common features in the convolutional layer, thus improving the training efficiency (Espejo-Garcia et al., 2022; Zhao X. et al., 2022). Inspired by this, this study is based on transfer learning to train the EfficientNet-B7-CBAM network.

All models utilized in this study were pretrained on the ImageNet dataset. The pre-trained weights were used only for initialization. All models were fully trained using the previously created plug seedling data. Due to the fact that there were only three types of plug seedlings, the final fully connected layer in each network was reduced from 1,000 to 3. SoftMax activation was implemented in the final layer. Using the Adam optimization algorithm and categorical cross-entropy as a loss, the models were trained. The Adam optimization algorithm’s parameters were as described as: α = 0.001, β_1_ = 0.9, β_2_ = 0.999, and ε = 10^–8^. There was a maximum of 300 iterations. The initial learning was set to 0.001, and the learning rate decayed to the original 0.8 for every 10 training epochs. The batch size was limited to 16 due to hardware limitations. We used dropout before the last layer of each model. A dropout rate of 0.45 was observed in this paper’s model.

## Experimental results and analysis

### Experimental configuration

Configuration of the hardware: GPU: GeForce GTX 1080Ti with 12 GB of video memory. The NVIDIA graphics drivers installed were CUDA 10.1 and CUDNNV7.6. It was NVIDIA’s GPU parallel computing framework that enabled users to solve complex computing problems using GPUs. CuDNN was a GPU accelerator developed by NVIDIA for deep neural networks. Windows 10 was the operating system of the software, and Python 3.8.5 was used to create the Pytorch deep learning framework and Opencv open-source visual library.

### Model evaluation index

The confusion matrix is an effective tool for evaluating the classification model’s merit and performance (Gajjar et al., 2022; Zhao Y. et al., 2022). Typically, the measures of model performance in the confusion matrix are Recall (*R*_*e*_), F1-Score (*F*_1_), Precision (*P*_*r*_), and Accuracy (*A*_*cc*_). The above formula for the four indexes can be expressed as Equations 5, 8.


(5) $$R_{e}=\frac{T\,P}{T\,P+F\,N}$$

(6) $$P_{r}=\frac{T\,P}{T\,P+F\,P}$$

(7) $$F_{1}=2\cdot \frac{P\,r\,e\,c\,i\,s\,i\,o\,n\times R\,e\,c\,a\,l\,l}{P\,r\,e\,c\,i\,s\,i\,o\,n+R\,e\,c\,a\,l\,l}$$

(8) $$A_{c\,c}=\frac{T\,P+T\,N}{T\,P+T\,N+F\,P+F\,N}$$

where *TP* represents the number of samples predicted by the model to be in a positive class that were actually in a positive class, whereas *FP* represents the number of samples predicted to be in a positive class that were actually in a negative class. *TN* is the number of samples predicted by the model to be in the negative class that are in the negative class. *FN* indicates the number of samples that the model predicted to be in a negative class but were actually in a positive class.

### Results and analysis

In this section, all models were validated on the re-collected data set of 450 unlabeled images of pepper plug seedlings (150 images of each plug seedling type). Concurrently, the evaluation index was the proposed confusion matrix from Section “Model evaluation index.”

#### Ablation experiments

In order to verify the effectiveness of the EfficientNet-B7-CBAM model, the following five abatement experiments were set up. (1) The original EfficientNet-B7 model. (2) In scheme 1 based on EfficientNet-B7 model trained using transfer learning, which constructed TL-EfficientNet-B7 model. (3) Used Adam’s optimization algorithm to train the TL-EfficientNet-B7 model, which constructed the TL-EfficientNet-B7-Adam model. (4) Replaced the SE module with the CBAM module in the TL-EfficientNet-B7-Adam model, which constructed the TL-EfficientNet-B7-Adam+SE- > CBAM model. (5) The EfficientNet-B7-CBAM model in this paper.

**FIGURE 11:**
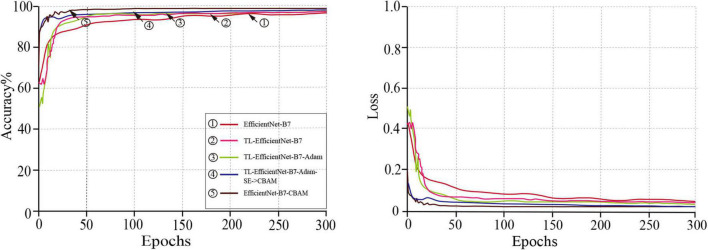
The training curves of the models.

The training results of the models for the five schemes described above are shown in Table 2. Compared to the experimental results of schemes 1 and 2, the average classification accuracy of the TL-EfficientNet-B7 model for plug seedlings reached 94.66%, which was 3.99% higher than that of the model in scheme 1. Additionally, the model’s training time was reduced by 23.5 min, and the transfer learning method effectively enhanced the model’s generalization ability. Compared to the experimental results of schemes 2 and 3, the average classification accuracy of the TL-EfficientNet-B7-Adam model was 95.33%, a 0.67% improvement over the scheme 2 models, and its training time was reduced by 20.7 min. The Adam optimization algorithm could hasten the model’s convergence and enhance its performance. The effectiveness of the scheme improvement was demonstrated by the fact that the overall accuracy of the TL-EfficientNet-B7-Adam model increased by 4.66%, and the training time was reduced by 44.2 min using both transfer learning and Adam optimization algorithms. In addition, experiments comparing schemes 3 and 4 demonstrated that the CBAM module possessed superior attention learning capability to the SE module. Compared to the experimental results of schemes 4 and 5, the addition of the CBAM module after the second convolutional layer improves the model’s ability to extract information. The experimental results shown in Table 2 demonstrated that the enhanced EfficientNet-B7-CBAM model achieved a classification accuracy of 97.99% on the previously constructed plug seedling dataset, which was 7.32% better than before the enhancement, and that the model training time was reduced by 60.2 min. The training curves of the models proposed by the five schemes are shown in Figure 11. According to Figure 11, the EfficientNet-B7-CBAM model converged around the 40th iteration, which was the quickest convergence speed of all models.

**TABLE 2:** Results of ablation experiment.

No.	Average *A*_*cc*_/%	Average *P*_*r*_/%	Average *R*_*e*_/%	Average *F*_1_/%	Times of training (min)
1	90.67	91.03	90.66	90.84	96.7
2	94.66	94.82	94.67	94.75	73.2
3	95.33	95.41	95.33	95.37	52.5
4	96.66	96.76	96.67	96.72	40.9
5	97.99	98.01	98.00	98.01	36.5

In combination with the above findings, the model training scheme’s feasibility and effectiveness could be determined. The improved EfficientNet-B7-CBAM model performed the classification of plug seedling quality with high accuracy and robustness.

#### The impact of data enhancement on model performance

Data augmentation was performed on the plug seedling images to increase the EfficientNet-B7-CBAM model’s resistance to interference in complex environments and prevent overfitting issues. A series of comparison experiments were designed to demonstrate the effect of data augmentation on model performance improvement in order to verify the effect of data augmentation. The experimental results before and after model data enhancement is shown in Table 3. By comparing the model’s *A*_*cc*_, *R*_*e*_, *P*_*r*_, and *F*_1_ performance metrics for each category on the plug seedling test set. On the test set, the classification recognition accuracy of the model trained on the original plug seedling data was 96.45%, which was 1.54% than the classification accuracy of the model after data enhancement. The experimental results demonstrated that data augmentation can improve model performance and contribute to the classification of plug seedlings.

**TABLE 3:** Performance of the model before and after data augmentation.

Data set	Class	*P*_*r*_/%	*R*_*e*_/%	*F*_1_/%	*Acc*/%
Original	Empty plug cells	95.45	98.00	96.71	96.45
	Weak seedlings	95.36	95.33	95.01	
	Strong seedlings	94.70	96.00	97.63	
Data augmentation	Empty plug cells	97.40	100.00	98.68	97.99
	Weak seedlings	97.31	96.67	96.99	
	Strong seedlings	99.32	97.33	98.31	

#### The performance comparison of different convolutional neural network model

Several classical CNN models AlexNet, VGG16, InceptionV3, ResNet50, and DenseNet121 were used to classify datasets of different quality cavity seedlings in order to demonstrate the efficacy of EfficientNet-B7-CBAM Mode. In addition, the performance was compared to the EfficientNet-B7-CBAM model. To ensure the fairness of the experiment, the above CNN models and EfficientNet-B7-CBAM Mode were trained using the same strategy and hardware configuration. The classification performance of several models is shown in Table 4.

**TABLE 4:** Performance comparison with other models.

Model	Average *A*_*cc*_/%	Average *P*_*r*_/%	Average *R*_*e*_/%	Average *F*_1_/%	Times of training (min)
AlexNet	77.94	78.92	78.67	78.79	80.9
VGG16	81.98	82.75	81.78	82.27	220.9
InceptionV3	85.60	86.24	85.55	85.89	60.3
ResNet50	88.92	82.93	88.89	85.91	48.5
DenseNet121	89.11	89.56	89.11	89.34	42.2
EfficientNet-B7-CBAM	97.99	98.01	98.00	98.01	36.5

As shown in Table 4, the EfficientNet-B7-CBAM model had the highest average classification accuracy of 97.99% on the test set of different quality pepper plug seedlings, which was 20.05, 16.01, 12.39, 9.07, and 8.88% higher than the average classification accuracy of a number of other models, respectively. Additionally, the EfficientNet-B7-CBAM model’s training time was only 36.5 min. In summary, the EfficientNet-B7-CBAM model had a significant advantage in terms of accuracy and training time, and was better able to meet the classification requirements for plug seedling quality.

#### Confusion matrix of the model

**FIGURE 12:**
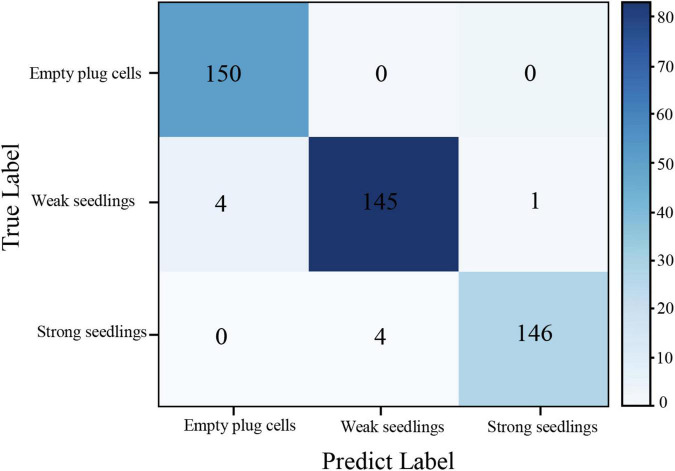
The EfficientNet-B7-CBAM model confusion matrix.

The confusion matrix of the EfficientNet-B7-CBAM model applied to the test set of plug seedlings, as shown in Figure 12. The average classification accuracy of the EfficientNet-B7-CBAM model for the three types of plug seedlings was 97.99%, the average *P*_*r*_ was 98.01%, the average *R*_*e*_ was 98.00%, and the overall index *F*_1_ was 98.01%, as determined by the confusion matrix. From the confusion matrix, it was evident that empty plug cells, weak seedlings, and Strong seedlings were misclassified as one another. Empty plug cells were misclassified as weak seedlings due to the presence of shed leaves; weak seedlings were misclassified as strong seedlings due to the interference of leaves protruding from seedlings in adjacent cells; and strong seedlings were misclassified as weak seedlings due to the incorrect angle of the plug seedlings and the low resolution of the images in this category.

## Conclusion

In order to support effective management of seedlings, an improved convolutional neural network with an attention mechanism was proposed in this work. The device acquisition system was used to collect a total of 8,109 images of plug seedlings for the model training process. Image augmentation was used to expand the dataset during the data preparation stage. The original EfficientNet-B7 model and the CBAM module were thoroughly integrated to acquire feature channels and spatial location data simultaneously. To hasten the model’s convergence, a transfer learning technique and the Adam optimization algorithm were also applied. The suggested model underwent extensive training, testing, and comparative experimentation. The proposed method in this study reaches recognition accuracy of 97.99%, which is better than other deep learning techniques currently in use, according to experimental results. The method’s competitive performance on the task of classifying the quality of plug seedlings served as a benchmark for the use of deep learning techniques in plug seedling classification. Our follow-up studies aim to expand the dataset and enhance the model’s ability to generalize in challenging situations. Additionally, by quantizing and pruning the model to reduce the number of parameters, and speed up the model convergence.

## Data availability statement

The raw data supporting the conclusions of this article will be made available by the authors, without undue reservation.

## Author contributions

XD: guidance on thesis writing and funding. LS: original writing. XJ: supervision, revision, and funding. PL: helped to set up the test bench and organize the data. ZY: helped to build the test bench and organize data. KG: helped to build the testbed and organize the data. All authors contributed to the article and approved the submitted version.
